# Left ventricular longitudinal wall fractional shortening accurately predicts longitudinal strain in critically ill patients with septic shock

**DOI:** 10.1186/s13613-021-00840-6

**Published:** 2021-03-30

**Authors:** Patrik Johansson Blixt, Michelle S. Chew, Rasmus Åhman, Lina de Geer, Lill Blomqwist, Meriam Åström Aneq, Jan Engvall, Henrik Andersson

**Affiliations:** 1 Department of Anaesthesiology and Intensive Care, Biomedical and Clinical Sciences, Linköping University, S-58185 Linköping, Sweden; 2 Department of Anaesthesiology and Intensive Care, Skane University Hospital, Malmö, Sweden; 3 Department of Clinical Physiology and Department of Health, Medicine and Caring Sciences, Linköping University, Linköping, Sweden; 4 Center of Medical Image Science and Visualization, Linköping University, Linköping, Sweden

**Keywords:** Strain, Fractional shortening, MAPSE, Left ventricle, Systolic function, Sepsis

## Abstract

**Background:**

Left ventricular longitudinal strain (LVLS) may be a sensitive indicator of left ventricular (LV) systolic function in patients with sepsis, but is dependent on high image quality and analysis software. Mitral annular plane systolic excursion (MAPSE) and the novel left ventricular longitudinal wall fractional shortening (LV-LWFS) are bedside echocardiographic indicators of LV systolic function that are less dependent on image quality. Both are sparsely investigated in the critically ill population, and may potentially be used as surrogates for LVLS. We assessed if LVLS may be predicted by LV-LWFS and MAPSE in patients with septic shock. We also assessed the repeatability and inter-rater agreement of LVLS, LV-LWFS and MAPSE measurements.

**Results:**

122 TTE studies from 3 echocardiographic data repositories of patients admitted to ICU with septic shock were retrospectively assessed, of which 73 were suitable for LVLS analysis using speckle tracking. The correlations between LVLS vs. LV-LWFS and LVLS vs. MAPSE were 0.89 (*p* < 0.001) and 0.81 (*p* < 0.001) with mean squared errors of 5.8% and 9.1%, respectively. Using the generated regression equation, LV-LWFS predicted LVLS with a high degree of accuracy and precision, with bias and limits of agreement of -0.044 ± 4.7% and mean squared prediction error of 5.8%. Interobserver repeatability was good, with high intraclass correlation coefficients (0.96–0.97), small bias and tight limits of agreement (≤ 4.1% for all analyses) between observers for all measurements.

**Conclusions:**

LV-LWFS may be used to estimate LVLS in patients with septic shock. MAPSE also performed well, but was slightly inferior compared to LV-LWFS in estimating LVLS. Feasibility of MAPSE and LV-LWFS was excellent, as was interobserver repeatability.

## Introduction

Left ventricular myocardial dysfunction occurs commonly in septic shock, yet its detection and association with clinical outcomes remains elusive. Traditional measurements such as left ventricular ejection fraction have not been associated with mortality [1–3] and are variably affected by the use of mechanical ventilation, vasopressors and inotropes [4].

Measurement of left ventricular global longitudinal strain (LVLS) using 2-D speckle tracking is an angle-independent method of measuring the function of the longitudinally oriented subendocardial myocardial fibers. Measurement of left ventricular (LV) systolic function using LVLS seems to be more sensitive than LV ejection fraction (LVEF) in detecting myocardial dysfunction before the appearance of clinically identifiable changes [5–7]. A recent meta-analysis identified LVLS as a better prognostic indicator for mortality than LVEF in patients with sepsis [8]. However, these findings were supported by limited evidence and some studies are contradictory [3, 7, 9, 10].

LVLS measurement requires good acoustic windows and accurate definition of the myocardial wall throughout the cardiac cycle. This is particularly challenging in intensive care units where the feasibility of LVLS measurement is generally poor [11]. Furthermore, measurements are normally done offline limiting its availability as well as being highly software and manufacturer dependent [12, 13].

In a recent paper, Huang et al. [14] described a new method for estimating LVLS from mitral annular plane systolic excursion (MAPSE). The new method essentially normalizes MAPSE to the length of the left ventricle (VL), and is called left ventricular longitudinal wall fractional shortening (LV-LWFS). LV-LWFS is given by Eq. 1:

1 $$\mathrm{LV}-\mathrm{LWFS}= \frac{\mathrm{MAPSE}}{\mathrm{VL}}\times 100$$

where MAPSE is the average of lateral and medial measurement and VL is the average of lateral and medial left ventricular length. Similar approaches to approximate strain have been previously described using left atrium emptying fraction, demonstrating a strong correlation with left atrium longitudinal strain [15].

LV-LWFS was a reproducible and reliable estimator of LVLS in critically ill patients with excellent correlations between the two measurements. LV-LWFS performed well in an internal validation with good predictive ability for LVLS as well as good intra- and inter-rater repeatability [14].

One of the advantages of using MAPSE and LV-LWFS is that they are calculated from M-mode and 2-D images directly available on any standard echocardiographic scanner. They are also easily obtained in critically ill patients [16] being less dependent on high image quality, a key factor in mechanically ventilated patients and patients with limited acoustic windows. However, it is conceivable that these simpler echocardiographic parameters may not be as reliable in selected groups of patients such as those with sepsis. For instance, only a few studies have evaluated MAPSE in the critically ill population and its relation to LVLS in patients with septic shock has not been described. Since LVLS and MAPSE are now two out of the four recommended parameters by the European Society of Intensive Care Medicine (ESICM) to assess LV systolic function in the critical care setting, continuing investigations and comparisons are warranted [17]. In the paper by Huang et al. [14], measurements were made in a heterogeneous group of patients, and none with septic shock. Thus, the aim of the present study is to determine if MAPSE and LV-LWFS can be used as surrogate measures of LVLS by examining their correlation, dispersion and agreement, in a group of critically ill patients with septic shock. We will also evaluate our predicted LVLS model against a previous model [14] obtained in patients without septic shock.

## Methods

One hundred and twenty-two transthoracic echocardiography (TTE) studies were retrieved from 3 echocardiographic research data repositories of patients admitted to ICU with septic shock. These studies were planned as longitudinal studies of 135 patients admitted with septic shock according to the Surviving Sepsis Campaign Criteria (2004 and 2012) and ethical approval was obtained for each (Regional Ethics Review Board, Lund, Sweden: 187/2005, Regional Ethical Review Board in Linköping, Sweden: 2012/233-31 and 2016/361-31). Written, informed consent was obtained from all participants or their proxies (next of kin). Our study is reported according to the STROBE guidelines (supplementary file 1). The TTE studies were performed on a Hewlett-Packard Sonos 5500 Scanner with a 3 MHz transducer or a GE Vivid 9 scanner with a 1.5–4.5 MHz (M5S-D) transducer. All echocardiographic studies were conducted within 12 h of ICU admission by trained sonographers, clinical physiologists or ICU physicians with > 100 h of echocardiography experience. For patients on mechanical ventilation, recordings were made irrespective of the phase of respiration. At least 3 cardiac cycles were stored and used for analysis. All images were stored in DICOM format and imported into a common third-party program for image analysis in order to avoid inter-vendor differences for LVLS analysis (Tomtec 2D Cardiac Performance Analysis version 1.3.0.147).

Two assessors independently screened the images and assessed them for suitability of analysis. To be included for analysis, the a priori inclusion criteria were sinus rhythm, apical four-chamber view images of sufficient quality for endocardial border tracing (≥ 4 out of 6 segments visible) and a frame rate ≥ 30 FPS. The long axis of the left ventricle had to be close to the midline in order to allow proper 2D measurements of ventricular length.

LVLS was measured using speckle tracking from the A4C view. After optimizing clarity, the endocardial border was traced manually and the region of interest (ROI) was adjusted to exclude the pericardium, trabeculae and papillary muscles. Peak myocardial strain for each of the three lateral as well as the three medial segments was averaged manually. Three cardiac cycles were traced and averaged to calculate LVLS.

**Fig. 1:**
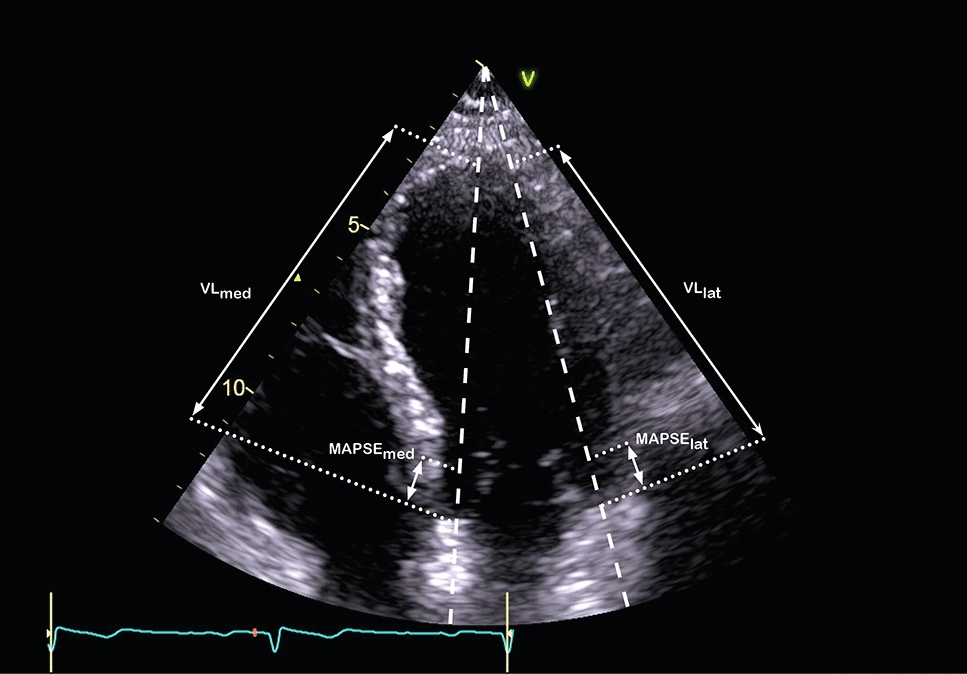
Measurement of LV-LWFS from the A4C view. MAPSE was calculated from an average of lateral and medial measurements. Lateral and medial ventricular length were measured directly from the 2D image and averaged. MAPSE_lat_ and MAPSE_med=_  = lateral and medial mitral annular plane systolic excursion, respectively, VL_lat_ and VL_med_ = lateral and medial left ventricular length, respectively

MAPSE was obtained from the displacement measurements in the Tomtec software. MAPSE was calculated as the average of lateral and medial mitral annular plane systolic excursion. Post-systolic shortening was avoided by gating the measurements to the ECG. MAPSE was calculated from an average of three cardiac cycles. VL was measured directly from the 2D image using a DICOM viewer (MicroDicom viewer 3.1.4), with length calibration performed for each image. VL was calculated as the average of lateral and medial 2D left ventricular lengths (Fig. 1). LV-LWFS was calculated using Eq. 1. LVEF was measured using the modified Simpson method (biplane method of disks).

All measurements were made by a single operator (PJB), and 70% of all images were assessed by a second operator (RÅ) for calculation of interobserver repeatability. Both operators had at least 40 h of training in strain measurement under the supervision of experienced ultrasonographers (MC, MÅA and JE).

## Statistics

Sample size was estimated using Green’s rule of thumb for a medium effect size ($n=50+(8 \times \text{numbers of predictors})$), yielding a minimum sample size of 58 [18]. Data are presented as median and interquartile range (IQR) or proportions (%) with 95% confidence intervals (95% CI) where applicable.

Correlations between LVLS and LV-LWFS or MAPSE were assessed using Pearson’s product moment correlation coefficient. To assess LV-LWFS or MAPSE as a predictor of LVLS, we used linear regression and examined residual error terms by calculating the mean squared error (MSE). We assessed the bias and precision of LV-LWFS vs LVLS using Bland–Altman plots.

Based on the linear regression equation, we calculated a predicted LVLS from LV-LWFS measurements. We compared measured LVLS (LVLS_meas_) with predicted LVLS using our model (LVLS_pred1_) and with predicted LVLS using a previous model defined by Huang et al. (LVLS_pred2_) [14], calculating the mean squared prediction error (MSPE) using Eq. 2:

2 $$\mathrm{MSPE}= \frac{\sum {({\mathrm{LVLS}}_{\mathrm{meas}}-{\mathrm{LVLS}}_{\mathrm{pred}1\mathrm{/pred}2})}^{2}}{n}$$

where n is the sample size, LVLS_meas_ is the measured value, LVLS_pred1_ is the predicted value using our model and LVLS_pred2_ is the predicted value using the model by Huang et al. [14]. The agreement between the two predicted LVLS values was assessed with Bland–Altman analysis and the intraclass correlation coefficient (ICC). LVLS was presented as absolute (positive) values according to recommendations [19].

In order to assess the interobserver repeatability of LV-LWFS measurements, we used the Bland–Altman method and ICC.

All analyses were made in IBM SPSS Statistics (ver 25.0, IBM Corp., Armonk, NY).

## Results

**Fig. 2:**
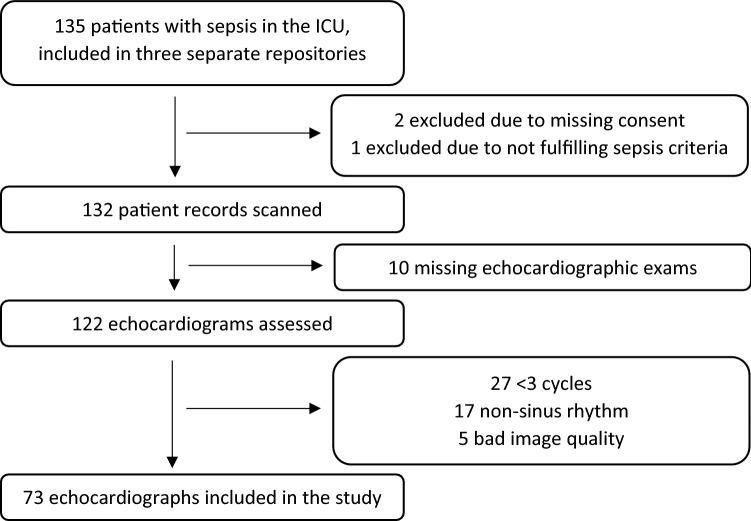
Flowchart showing details of included and excluded studies

Of the 135 patients in the database, 122 patients had echocardiographic studies available, of which 73 met the inclusion criteria (Fig. 2). None of the patients had mitral valve protheses, severe mitral valvular dysfunction, or tamponade physiology. Fifty-four percent of all patients had impaired LV systolic function defined as LVEF ≤ 50%. Characteristics of patients included in the study on the day of admission are shown in Table 1.

**Table 1:** Baseline patient characteristics on Day 1 of admission

	Study cohort *n* = 73
Age, median (IQR)	66 (54–76)
Weight, kg, median (IQR)	79 (65–85)
Male, *n* (%)	48 (66)
Preexisting cardiac disease*, *n* (%)	38 (52)
Apache II score, median (IQR)	20 (16–26)
SOFA score on admission, median (IQR)	11 (8–13)
Mechanically ventilated during echocardiography, *n* (%)	55 (75)
PEEP, cmH_2_O, median (IQR)	8 (7–12)
Mechanical ventilation days, median (IQR)	6 (3–13)
CRRT, *n* (%)	15 (21)
Vasopressors/inotropes during echocardiography, *n* (%)	65 (89)
hsTnT (ng/L), median (IQR)	79 (37–201)
ICU length of stay days, median (IQR)	7 (4–12)
ICU mortality, *n* (%)	15 (21)
30-day mortality, *n* (%)	18 (25)
Regional wall motion abnormality, *n* (%)	9 (12%)
% with LVEF ≤ 50%	54%
LVLS, %, median (IQR)	− 15.0 (− 10.7–− 18.6)
MAPSE, mm, median (IQR)	10.3 (7.2–13.0)
LWFS, %, median (IQR)	11.7 (8.3–14.2)

*Defined as arrhythmia, heart failure or ischaemic heart disease

*SOFA* Sequential Organ Failure Assessment, *PEEP* positive end-expiratory pressure, *CRRT* continuous renal replacement therapy; *hsTnT* high-sensitivity Troponin T, *ICU* intensive care unit, *LVEF* left ventricle ejection fraction; *LVLS* left ventricle longitudinal strain, *MAPSE* mitral annular plane systolic excursion, *LV-LWFS* left ventricle-longitudinal wall fractional shortening

### LV-LWFS and MAPSE correlate with LVLS

There were good correlations between LVLS and LV-LWFS as well as LVLS and MAPSE (*p* < 0.001 for both). The limits of agreement (LOA) for LVLS vs LV-LWFS were 4.8%. The Pearson moment correlation coefficients and corresponding MSEs are shown in Table 2.

**Table 2:** Linear regression (including 95% CI), Pearson’s product moment correlation coefficient, MSE, bias ± LOA between LVLS, MAPSE and LV-LWFS

	Pearson’s *r* (95% CI)	MSE (%)	Bias (LOA, %)
y = 1.086x + 2.012	0.89 (0.83–0.92)	5.8	− 3.0 (± 4.8)
y = 1.100x + 3.296	0.81 (0.74–0.87)	9.1	NA*

*Bland–Altman analysis was not possible since MAPSE and LVLS are measured in different units

*CI* confidence interval, *MSE* mean square error, *LOA* limits of agreement, *LVLS* left ventricle longitudinal strain, *MAPSE* mitral annular plane systolic excursion, *LV-LWFS* left ventricle-longitudinal wall fractional shortening

### Predicted LVLS from LV-LWFS measurements in patients with septic shock compares well to previous prediction model in non-septic shock patients

In order to compare the predictive capability of LV-LWFS for LVLS derived from different populations, we compared the predicted values using the regression equation from our cohort of septic shock patients (LVLS_pred1_) with the predicted values obtained using the regression equation from Huang et al. (LVLS_pred2_) [14]. LVLS_pred_ calculated from both prediction models demonstrated excellent correlation with measured LVLS, with similar regression equations and *r*^2^ values, regardless of the model chosen. MSPEs were small (5.8% for LVLS_pred1_ and 5.8% for LVLS_pred2_) as were the LOA (Table 3).

**Table 3:** Linear regression (including 95% CI), Pearson’s product moment correlation coefficient, MSPE, bias ± LOA, and ICC for measured vs predicted LVLS

	MSPE	Bias (LOA)	ICC (95% CI)
y = 0.997x + 0.007	5.8	− 0.044 (± 4.7%)	0.94 (0.90–0.96)
y = 0.921x + 2.691	5.8	1.649 (± 4.8%)	0.94 (0.90–0.96)

*CI* confidence interval, *MSPE* mean square prediction error, *LOA* limits of agreement, *ICC* intraclass correlation coefficient, *LVLS* left ventricle longitudinal strain, *LV-LWFS* left ventricle-longitudinal wall fractional shortening, *LVLSmeas* measured LVLS, *LVLSpred1* predicted LVLS from LV-LWFS using the regression equation from our cohort, *LVLSpred2* predicted LVLS from LV-LWFS using the regression equation from Huang et al. [14]

### Interobserver repeatability

Bland–Altman analysis was used to assess the interobserver agreement between LV-LWFS measurements made by two independent assessors, on the same images. This revealed only minimal bias and small LOA (− 0.484 ± 3.3%), and high ICC 0.96 (0.94–0.98). Interobserver agreements for LVLS and MAPSE were 0.234 (± 4.1%) and 0.445 (± 3.0 mm), and their corresponding ICCs were 0.97 (0.94–0.98) and 0.96 (0.94–0.98).

## Discussion

In this cohort of patients with septic shock, we confirm that LV-LWFS may be used to accurately estimate LVLS. The MSEs were small indicating that there is only a small dispersion from the regression lines. There was good agreement between LVLS and LV-LWFS. Interrater repeatability of LV-LWFS assessed using Bland–Altman plots show a very small bias and tight LOA between the two independent observers, supported by a high ICC indicating that the method is reproducible. The performance of LV-LWFS as an estimator of actual LVLS was superior to MAPSE which also provided acceptable values. For comparison, LV-LWFS and MAPSE showed poor-to-average agreement with LVEF and s’, even if statistically significant (data not shown).

We also demonstrate that LV-LWFS may be used to estimate LVLS when using a previous regression equation in an ICU population without septic shock. Notably, the regression equation obtained in our cohort consisting only of patients with septic shock was remarkably similar to that described by Huang et al. that investigated a more heterogenous ICU population, without septic shock [14]. Impaired LV systolic function, defined as LVEF ≤ 50% was more common in our cohort, occurring in 54% of patients compared to 41% in Huang et al. [14]. The predictive ability of LV-LWFS was supported by small MSPEs when using both equations. Thus, we provide support for the use of LV-LWFS, a simpler measure of LV systolic function, for bedside estimation or where LVLS is not available or feasible.

We extend previous findings by external validation of the LV-LWFS method in a septic shock population. These data support the robustness of using LV-LWFS and MAPSE as surrogates for LVLS measurement. LV-LWFS and MAPSE are less dependent on image quality and may be especially useful in critically ill patients in whom acceptable image quality may be difficult to obtain. The feasibility of LV-LWFS assessment was excellent. Of the 122 echocardiograms available for assessment, 73 could be analyzed for LV-LWFS and LVLS measured from a single A4C view after exclusion for inadequate number of cardiac cycles and poor image quality. In comparison, only 26 echocardiograms (21%) had the required 3 views for standard GLS measurement (A4C, apical two chamber and apical long axis). Although measuring strain from a single view is a limitation of the present study, measurement of left ventricular strain from the A4C view is strongly correlated with the 3-view GLS and more pragmatic and feasible in the ICU setting [20]. Of the 122 studies that were available, feasibility was 100% for MAPSE and LV-LWFS, 90% for LVLS, 77% for GLS, 82% for biplane LVEF and 94% for s’. Thus, in ICUs where LVLS measurements are unavailable, or where bedside assessments are required, we suggest that MAPSE or LV-LWFS be used for estimating LVLS.

There were several limitations to the present study. We relied on echocardiographic studies from an existing data repository that was not designed for our purpose. We did not assess how loading conditions may have influenced the performance of the various echocardiographic parameters. However, all patients were included to ICU with defined septic shock criteria, were volume resuscitated and treated according to the Surviving Sepsis Guidelines and echocardiography was conducted within 12 h of admission.

The limitations of angle-dependency and only segmental information should be borne in mind. Although LV-LWFS and LVLS are related since both measure myocardial motion in the longitudinal plane, it only captures longitudinal motion at the level of the atrioventricular plane. For example, circumferential motion, measured with strain analysis, may compensate for a reduced longitudinal motion and hence preserve LVEF [21]. However, MAPSE, despite its simplicity, is considered to be the principal contributor to LVEF, accounting for at least 60% of stroke volume [22] and is more sensitive in detecting LV dysfunction than EF in patients with hypertension [23, 24] and does not seem to be inferior to global longitudinal strain measurements [25]. In another study, MAPSE and global longitudinal strain demonstrated similar biological variability [26], this variability was not improved by normalizing MAPSE to ventricular length. We did not investigate if LVLS, MAPSE, LV-LWFS or predicted LVLS values are associated with clinical outcome. Since recent studies suggest that MAPSE may be a useful predictor of clinical outcome [27, 28], this would be a logical line of investigation for future studies, along with further comparisons with commonly used parameters such as LVEF and s’.

## Conclusions

We provide an external validation of LV-LWFS for estimating LVLS in a septic shock population. MAPSE was also able to estimate LVLS, but with slightly poorer performance compared to LV-LWFS. Both MAPSE and LV-LWFS demonstrated excellent feasibility and interobserver repeatability. LV-LWFS agreed well with LVLS, demonstrating good accuracy and precision.

## Data Availability

The data from this study will be made available after publication, upon application to the corresponding author and within the terms of the Global Data Protection Regulation and the Swedish Patient Data Law (2008:355). To avoid the possibility of identifying individual cases, detailed data are not given in the paper but may be requested from the corresponding author.

## Abbreviations

LVLS: Left ventricular longitudinal strain
LV: Left ventricle
LVEF: Left ventricle ejection fraction
MAPSE: Mitral annular plane systolic excursion
VL: Ventricle length
LV-LWFS: Left ventricle longitudinal wall fractional shortening
TTE: Transthoracic echocardiography
FPS: Frames per second
ROI: Region of interest
IQR: Interquartile range
CI: Confidence interval
MSE: Mean square error
MSPE: Mean square prediction error
ICC: Intraclass correlation coefficient
SOFA: Sequential organ failure assessment
PEEP: Positive end-expiratory pressure
CRRT: Continuous renal replacement therapy
hsTnT: High-sensitivity Troponin T
ICU: Intensive care unit
LOA: Limits of agreement
LVLSmeas: Measured LVLS
LVLSpred1: Predicted LVLS from LV-LWFS using the regression equation from our cohort
LVLSpred2: Predicted LVLS from LV-LWFS using the regression equation from Huang et al. (14)
